# Nutrition management and self-perceived relapse triggers in patients with inflammatory bowel disease: results from the 3R web-based survey

**DOI:** 10.1093/crocol/otag081

**Published:** 2026-07-16

**Authors:** Motohiro Esaki, Takayuki Matsumoto, Fumihito Hirai, Takahiro Suzuki, Yoshiyuki Hosoi, Akira Oota, Masaaki Higashikawa, Minami Umeyama, Masato Ueno

**Affiliations:** Division of Gastroenterology, Department of Internal Medicine, Faculty of Medicine, Saga University, Saga, Japan; Division of Gastroenterology, Department of Internal Medicine, School of Medicine, Iwate Medical University, Iwate, Japan; Department of Gastroenterology, Faculty of Medicine, Fukuoka University, Fukuoka, Japan; Medical Department, EA Pharma, Tokyo, Japan; Medical Department, EA Pharma, Tokyo, Japan; Pharmaceutical Development Department, Data Science Group, EA Pharma, Tokyo, Japan; Pharmaceutical Development Department, Data Science Group, EA Pharma, Tokyo, Japan; Medical Department, EA Pharma, Tokyo, Japan; Medical Department, EA Pharma, Tokyo, Japan

**Keywords:** Crohn’s disease, ulcerative colitis, enteral nutrition, adherence, self-perceived relapse

## Abstract

**Background and Aims:**

Inflammatory bowel disease (IBD), which includes Crohn’s disease (CD) and ulcerative colitis (UC), requires long-term management. Besides pharmacotherapy, nutritional care is considered to be important; however, its real-world status in Japan remains unclear.

**Methods:**

In this study, a web-based survey on 150 patients with IBD (CD, 75; UC, 75) was conducted between October 30 and November 4, 2025. Patient demographics, self-perceived relapse frequency, self-perceived relapse triggers, implementation of nutritional therapy, and the use of health management apps were evaluated.

**Results:**

Patients with CD reported a higher self-perceived relapse frequency than did patients with UC (≥1 episode/year: 52% vs 32%) and were more likely to perceive diet as a self-perceived relapse trigger (77% vs 39%). Nutritional therapy was introduced in 53% of patients with CD and 12% of patients with UC, and 31% of patients with CD were currently using elemental diets. Most patients consumed 300-900 kcal/day, and some reported symptom improvement, although discontinuation was often due to the physician’s decision or difficulty in intake. Dietary management awareness was higher in the CD group than in the UC group (77% vs 48%), whereas supplement use showed no significant difference (37% vs 29%). Health app usage was <10%, with common reasons for nonuse being a lack of awareness and perceived necessity.

**Conclusions:**

Nutritional therapy is more frequently administered to patients with CD in Japan, suggesting its clinical relevance. Strategies are required to enhance dietary guidance, support the continued use of elemental diets, and promote digital health tools.

## Graphical abstract

**Graphical Abstract:**
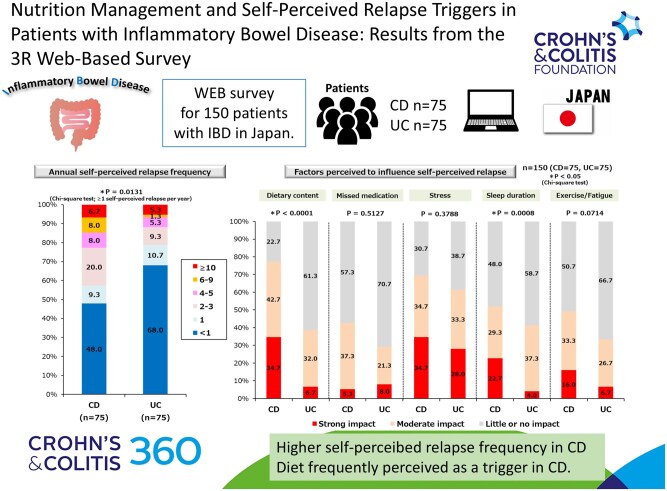

## Introduction

Inflammatory bowel diseases (IBD), including Crohn’s disease (CD) and ulcerative colitis (UC), are chronic relapsing conditions characterized by repeated cycles of remission and relapse.^1^ Pharmacological therapy remains the mainstay of long-term management; however, the importance of nutritional care, including dietary therapy and enteral nutrition, has been emphasized.^2–5^ Particularly, the elemental diet (ED) has been reported to reduce intestinal burden and potentially contribute to the control of inflammation.^6–10^ Nevertheless, with the increasing availability of new therapeutic agents,^11^ the current real-world status of nutritional management among Japanese patients with IBD, including the factors that patients perceive as influencing relapse, remains unclear.

Moreover, patients’ awareness of dietary management, use of supplements, and adoption of digital tools such as health management applications may influence treatment adherence and lifestyle modifications. However, clinical data on these aspects are limited. Understanding these factors is essential for optimizing patient education and treatment strategies.

Therefore, in this study, a web-based survey was conducted—the 3R survey (*R*eal-world patients survey about nutrition and *r*elation to *r*elapse of IBD)—to clarify the current status of nutritional therapy implementation and continuation, self-perceived relapse triggers, dietary management awareness, supplement use, and utilization of health management applications among Japanese patients with IBD.

## Methods

### Survey instrument

This was a cross-sectional web-based survey targeting patients with IBD in Japan. The survey was approved by the Institutional Ethics Committee and registered in the UMIN Clinical Trials Registry (UMIN-ID: 000059440). It was conducted by Cross Marketing Co., Ltd., under the commission of EA Pharma Co., Ltd. Between October 30 and November 4, 2025, invitations were sent via the internet to 62,521 individuals registered in a nationwide large-scale survey panel. The target sample size was set at 75 patients each with CD and UC, based on the estimated number of cases that could be recruited from the panel. Enrollment was terminated when the target number for each disease group was reached.

Survey items included patient demographics (age, sex, disease duration, body mass index [BMI], smoking history, history of intestinal resection, and medications), annual self-perceived relapse frequency, factors perceived to influence self-perceived relapse (dietary content, missed medication, stress, sleep duration, exercise/fatigue), experience of nutritional therapy introduction, current use and daily intake of EDs, perceived effects and reasons for discontinuation, awareness of dietary management, supplement use, and utilization of health management applications, including reasons for nonuse. Annual self-perceived relapse frequency was assessed using a single self-reported survey item. Participants were asked: “Approximately how many times have your symptoms worsened (relapsed) in the past year?”. Responses were recorded as the number of symptom worsening episodes over the preceding 12 months and were analyzed as a measure of self-perceived relapse.

### Statistical analysis

All statistical analyses were performed using the SAS software (version 9.4; SAS Institute Japan Ltd.). Categorical variables were analyzed using Pearson’s chi-square test. In addition, multivariable logistic regression analyses were performed as exploratory analyses to adjust for potential confounding factors. Odds ratios (ORs) and 95% confidence intervals (CIs) were calculated. Statistical significance was set at *P* < .05.

### Ethical considerations

This survey was approved by the Ethics Committee of the Yamauchi Clinic (Approval No. 2025-10-00377). The study was conducted in accordance with the Declaration of Helsinki, and informed consent for participation was obtained electronically via the Internet.

## Results

### Patient characteristics

Responses were obtained from 150 patients with IBD (75 with CD and 75 with UC) who consented to participate and publish the results. No responses were deemed ineligible; therefore, all patients were included in the analysis. The mean age was 50.9 years for CD and 55.3 years for UC, and the mean disease duration was 19.0 years and 14.5 years, respectively. The proportion of male patients was 69.3% in the CD group and 81.3% in the UC group (Table 1). Among all participants, the use of advanced therapy (ADT), including biologics and small-molecule agents, was reported in 53.3% of patients with CD (40/75) and 16.0% of patients with UC (12/75).

**Table 1:** Patient characteristics.

Variable	CD (*n* = 75)	UC (*n* = 75)
**Male (%)**	69.3% (*n* = 52)	81.3% (*n* = 61)
**Age, mean ± SD (years)**	50.9 ± 12.5	55.3 ± 10.7
**Disease duration, mean ± SD (years)**	19.0 ± 12.7	14.5 ± 8.9
**BMI, mean ± SD**	21.7 ± 4.8	22.7 ± 3.5
**Occupation**	Full-time: 49.3% (*n* = 37)	Full-time: 56.0% (*n* = 42)
	Part-time: 20.0% (*n* = 15)	Part-time: 17.3% (*n* = 13)
	Student: 1.3% (*n* = 1)	Student: 0%
	Homemaker: 5.3% (*n* = 4)	Homemaker: 4.0% (*n* = 3)
	Other: 5.3% (*n* = 4)	Other: 5.3% (*n* = 4)
	Unemployed: 18.7% (*n* = 14)	Unemployed: 17.3% (*n* = 13)
**Smoking history**	Never: 61.3% (*n* = 46)	Never: 48.0% (*n* = 36)
	Current: 18.7% (*n* = 14)	Current: 22.7% (*n* = 17)
	Former: 20.0% (*n* = 15)	Former: 29.3% (*n* = 22)
**History of intestinal resection**	49.3% (*n* = 37)	16.0% (*n* = 12)
**Number of hospitalizations, mean ± SD**	7.0 ± 11.6	1.4 ± 2.1
**Symptoms (diarrhea, abdominal pain, bloody stool)**	None: 33.3% (*n* = 25)	None: 49.3% (*n* = 37)
	Mild: 34.7% (*n* = 26)	Mild: 41.3% (*n* = 31)
	Moderate: 28.0% (*n* = 21)	Moderate: 9.3% (*n* = 7)
	Severe: 4.0% (*n* = 3)	Severe: 0%
**Type of medical institution**	University hospital: 38.7% (*n* = 29)	University hospital: 22.7% (*n* = 17)
	General hospital: 48.0% (*n* = 36)	General hospital: 53.3% (*n* = 40)
	Clinic: 10.7% (*n* = 8)	Clinic: 24.0% (*n* = 18)
	No regular visits: 2.7% (*n* = 2)	No regular visits: 0%
**Medications**	Oral mesalazine: 70.7% (*n* = 53)	Oral mesalazine: 81.3% (*n* = 61)
	Oral steroids: 9.3% (*n* = 7)	Oral steroids: 6.7% (*n* = 5)
	Carotegrast methyl: -	Carotegrast methyl: 1.3% (*n* = 1)
	Thiopurines: 10.7% (*n* = 8)	Thiopurines: 6.7% (*n* = 5)
	Infliximab: 20.0% (*n* = 15)	Infliximab: 1.3% (*n* = 1)
	Adalimumab: 10.7% (*n* = 8)	Adalimumab: 1.3% (*n* = 1)
	Golimumab: -	Golimumab: 1.3% (*n* = 1)
	Ustekinumab: 16.0% (*n* = 12)	Ustekinumab: 1.3% (*n* = 1)
	Vedolizumab: 0%	Vedolizumab: 1.3% (*n* = 1)
	Tofacitinib: -	Tofacitinib: 0%
	Filgotinib: -	Filgotinib: 2.7% (*n* = 2)
	Upadacitinib: 1.3% (*n* = 1)	Upadacitinib: 0%
	Mirikizumab: 0%	Mirikizumab: 1.3% (*n* = 1)
	Risankizumab: 1.3% (*n* = 1)	Risankizumab: 5.3% (*n* = 4)
	Guselkumab: 1.3% (*n* = 1)	Guselkumab: 2.7% (*n* = 2)
	Ozanimod: -	Ozanimod: 0%
	Etrasimod: -	Etrasimod: 0%
	Suppository: 2.7% (*n* = 2)	Suppository: 9.3% (*n* = 7)
	Enema solution: 1.3% (*n* = 1)	Enema solution: -
	Enema foam: 4.0% (*n* = 3)	Enema foam: -
	Elemental diet: 44.0% (*n* = 33)	Elemental diet: 0%

Abbreviation: SD, standard deviation.

### Self-perceived relapse frequency and perceived influencing factors

**Figure 1:**
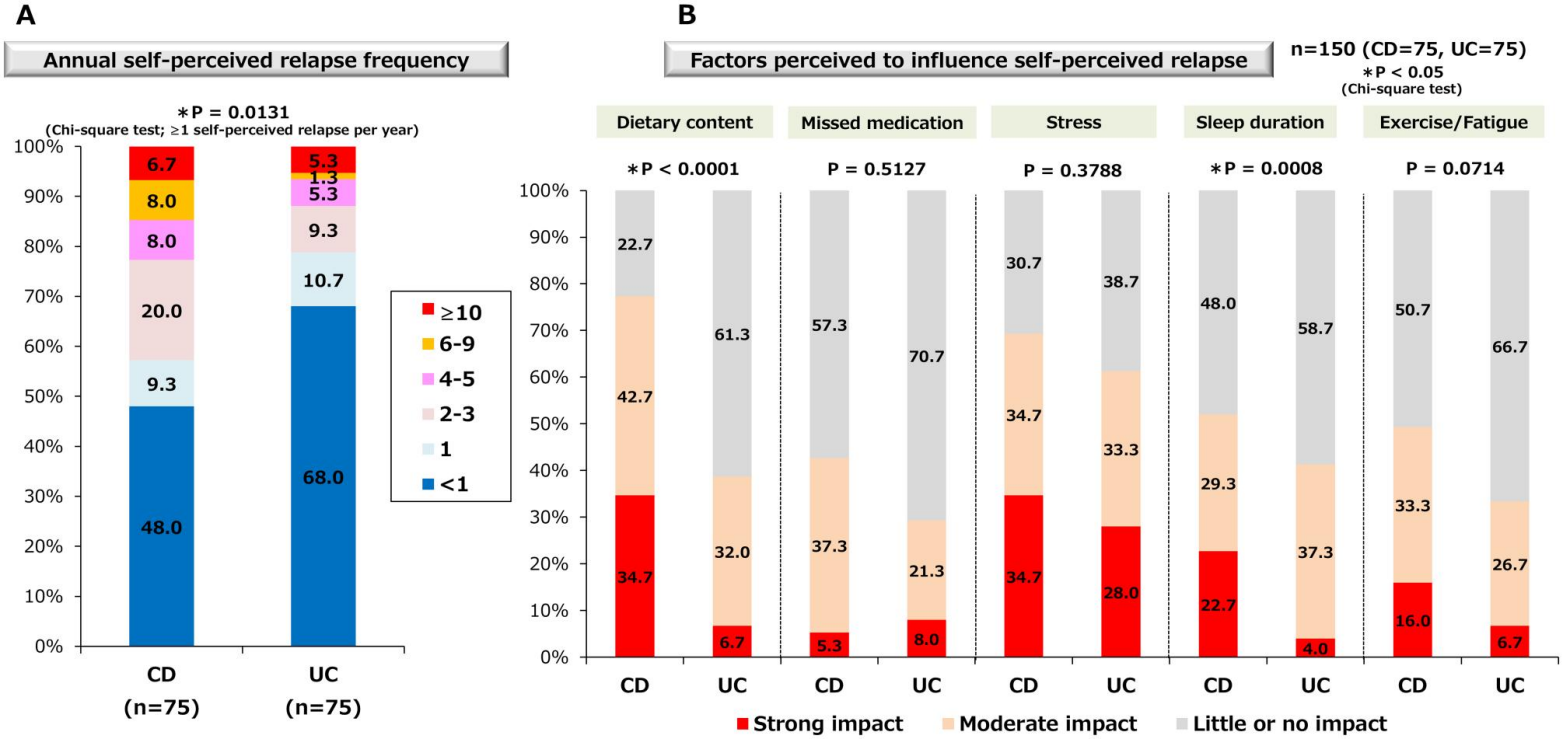
Self-perceived relapse frequency and perceived influencing factors. (A) Annual self-perceived relapse frequency in Crohn’s disease (CD) and ulcerative colitis (UC). Patients were categorized according to the number of self-perceived relapses experienced in the previous year (<1, 1, 2-3, 4-5, 6-9, ≥10 episodes). The proportion of patients reporting ≥1 self-perceived relapse per year was significantly higher in CD than in UC (*P* = .0131; chi‑square test). (B) Factors perceived to influence self-perceived relapse. Participants evaluated the impact of dietary content, missed medication, sleep duration, stress, and exercise/fatigue using three categories (“strong impact,” “moderate impact,” and “little or no impact”). Patients with CD more frequently reported a strong impact of diet and sleep compared with UC patients (*P* < .0001 and *P* = .0008, respectively). CD, Crohn’s disease; IBD, inflammatory bowel disease; UC, ulcerative colitis.

The proportion of patients reporting ≥1 self-perceived relapse per year was significantly higher in CD than in UC (52.0% vs 32.0%, *P* < .0131) (Figure 1A). This result remained significant under multivariable analysis adjusting for age and sex, with an OR of 2.254 in CD compared to UC (95% CI: 1.153-4.480, *P* = .0186). Regarding factors perceived to influence self-perceived relapse, 34.7% and 42.7% of patients with CD answered that dietary content had a “strong” or “moderate” impact, compared with 6.7% and 32.0% of patients with UC, respectively (*P* < .0001) (Figure 1B). Similarly, the proportion of patients who felt that sleep duration had a “strong impact” was significantly higher in the CD group than in the UC group (22.7% vs 4.0%, respectively, *P* = .0008). Regarding stress, many patients in both groups reported a strong impact, but no significant difference was observed between patients with CD and UC (34.7% vs 28.0%, *P* = .3788). After adjustment for disease type, sex, and disease duration, dietary content and sleep duration were strongly associated with self-perceived relapse in CD, with ORs of 6.793 (95% CI: 2.590-21.364, *P* = .0003) and 6.783 (95% CI: 2.112-30.372, *P* = .0036), respectively.

### Implementation of nutritional therapy

**Figure 2:**
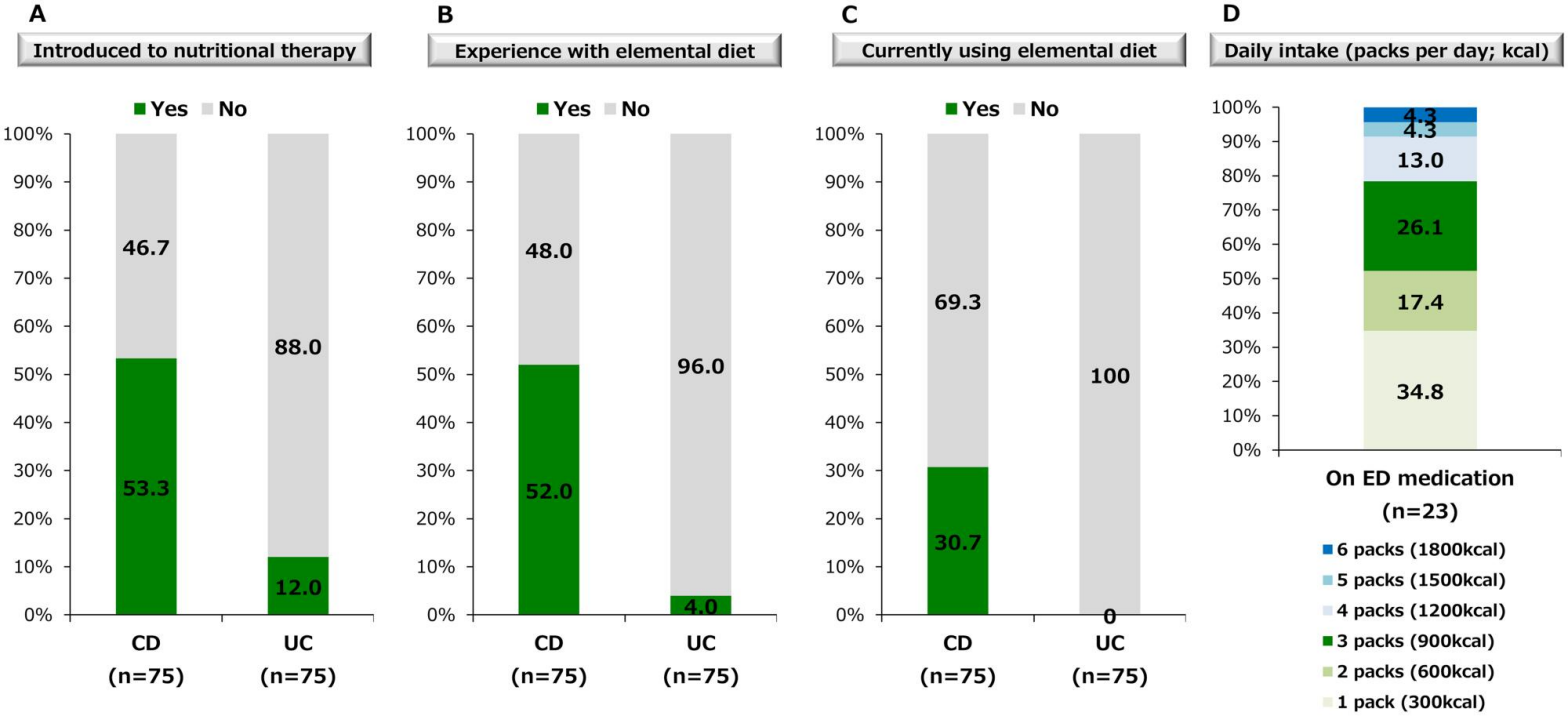
Implementation status of nutritional therapy. This figure summarizes the introduction of nutritional therapy, experiences with the ED, current ED use, and daily ED caloric intake. (A) Introduction to nutritional therapy (yes/no) according to disease group (CD vs UC). (B) Experience with the ED (ever/never) by disease group. (C) Current ED use at the time of the survey (yes/no) by disease group. (D) Daily ED intake among current users, shown as packs per day with approximate calories per pack (1 pack ≈ 300 kcal; categories: 1, 2, 3, 4, 5, 6 packs per day). Group comparisons were performed using the chi-square test, as appropriate. Bars depict percentages with the corresponding denominators shown on the x-axis. CD, Crohn’s disease; ED, elemental diet; UC, ulcerative colitis.

**Figure 3:**
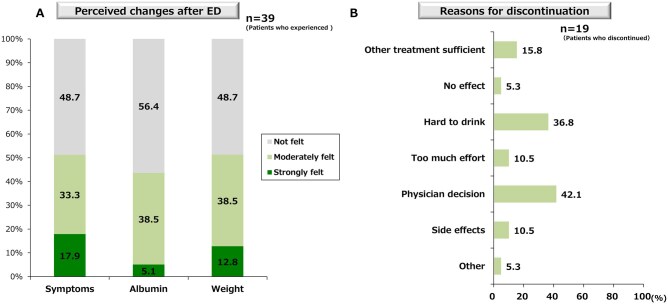
Perceived changes after elemental diet use and reasons for discontinuation. (A) Perceived changes after ED among patients with experience of ED (*n* = 39). Respondents rated improvements in gastrointestinal symptoms, serum albumin, and body weight on a 3‑point scale (“strongly felt,” “moderately felt,” “not felt”). Results are shown as percentages of respondents for each domain. (B) Reasons for discontinuation of ED among those who had stopped ED (*n* = 19). Reasons included physician’s decision, difficulty in intake, side effects, other treatments sufficient, no effect, and other. Multiple responses were allowed where applicable; bars indicate the proportion endorsing each reason. This analysis is descriptive and exploratory; no multiplicity adjustments were applied. ED, elemental diet.

Experience of nutritional therapy introduction was reported by 53.3% of patients with CD and 12.0% of patients with UC; 30.7% of patients with CD were currently taking EDs, whereas none in the UC group were (Figure 2). Among patients with CD, the proportion of those currently using an ED was 42.5% (17/40) in the ADT-user group and 17.1% (6/35) in the non-ADT group. No statistical comparison was performed because the analysis was exploratory and the sample size was limited. Among patients with CD, the daily ED intakes were 300 kcal (34.8%), 600 kcal (17.4%), and 900 kcal (26.1%). Self-assessment among patients with experience of ED use indicated symptom improvement was “strongly felt” by 17.9% and “moderately felt” by 33.3%; improvement in albumin was reported by 5.1% and 38.5%, and weight gain by 12.8% and 38.5%, respectively (Figure 3A). Common reasons for discontinuation included “physician’s decision” (42.1%) and “difficulty in intake” (36.8%) (Figure 3B).

### Daily nutritional management

**Figure 4:**
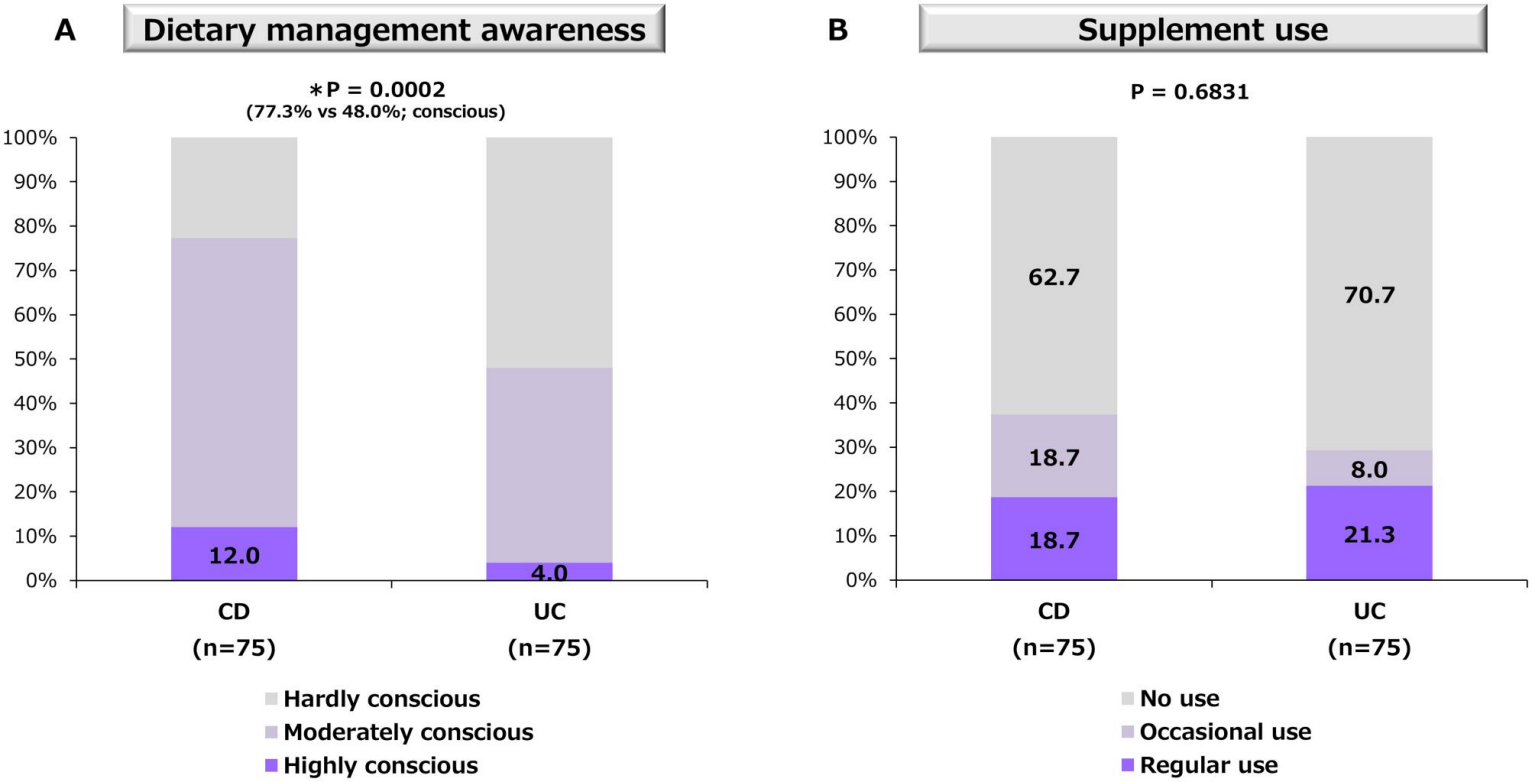
Dietary management and supplement use. (A) Awareness/practice of dietary management by disease group (CD vs UC), categorized as highly conscious, moderately conscious, and hardly conscious. The proportion of patients conscious of dietary management (highly or moderately) was higher in CD than in UC (77.3% vs 48.0%; *P* = .0002; chi‑square test). (B) Use of nutritional supplements, categorized as regular use, occasional use, and no use by disease group. Between‑group differences were not statistically significant (CD 37.3% vs UC 29.3% for any use). Bars represent percentages; denominators are shown on the x‑axis. CD, Crohn’s disease; UC, ulcerative colitis.

The proportion of patients who consciously managed their dietary content was significantly higher in the CD group than in the UC group (77.3% vs 48.0%, *P* = .0002) (Figure 4A). Overall supplement use was reported by 37.3% of patients with CD and 29.3% of patients with UC, with no significant difference between the groups (Figure 4B).

### Medication adherence

**Figure 5:**
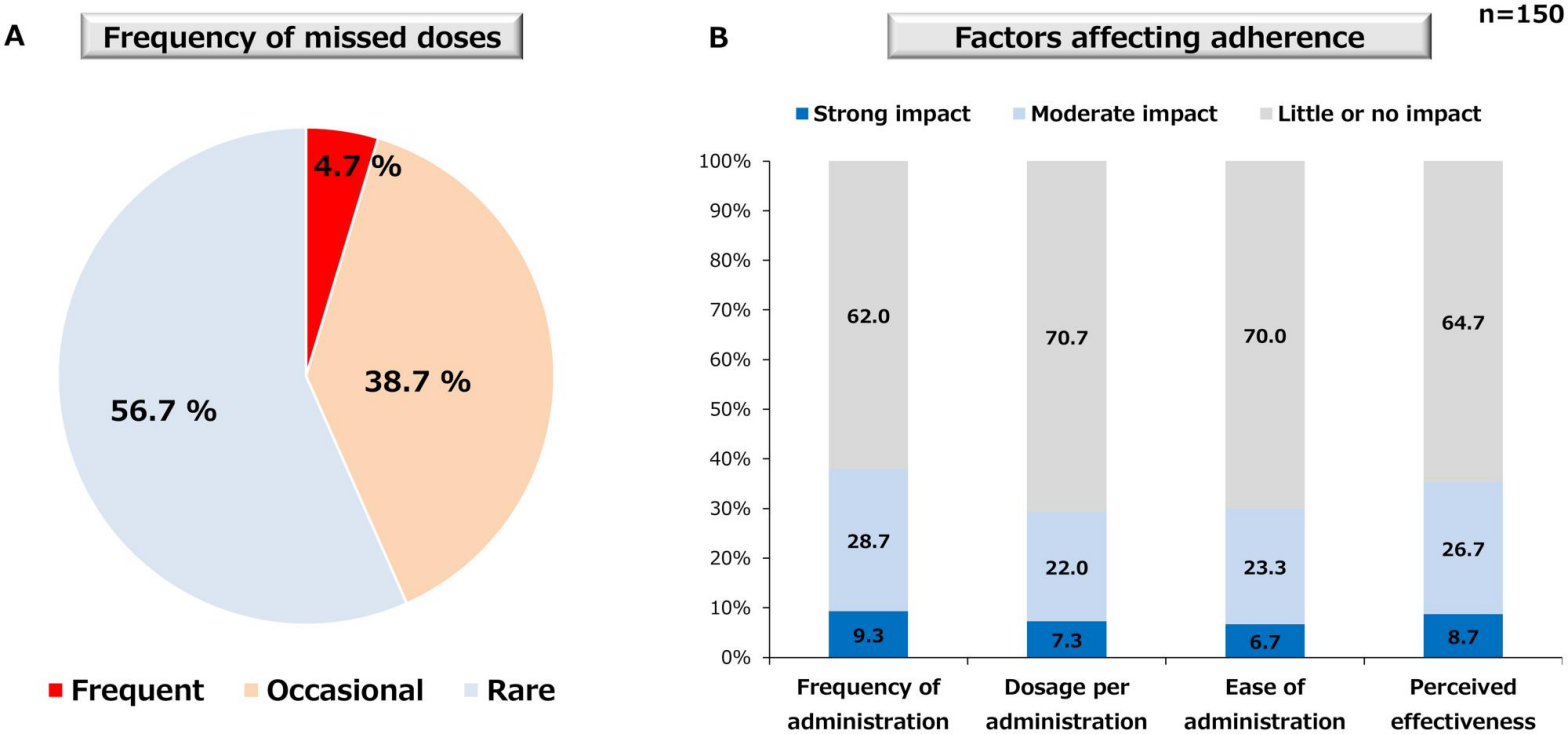
Medication adherence and influencing factors. (A) Frequency of missed doses in the whole cohort (*n* = 150), categorized as frequent, occasional, or rare. (B) Factors perceived to affect adherence, including frequency of administration, dose per administration, ease of administration, and perceived effectiveness. Each factor was rated on a 3‑point scale (“strong impact,” “moderate impact,” “little or no impact”), and results are shown as percentages. This figure presents descriptive patient-reported outcomes. No formal between-group hypothesis testing was planned for Panel B.

Regarding medication adherence, 4.7% of patients reported frequent missed doses and 38.7% reported occasional missed doses (Figure 5A). Factors perceived to influence adherence included “frequency of administration,” “dosage,” “ease of administration,” and “perceived effectiveness,” each reported by approximately 30% of patients (Figure 5B).

### Use of health and nutrition management applications

**Figure 6:**
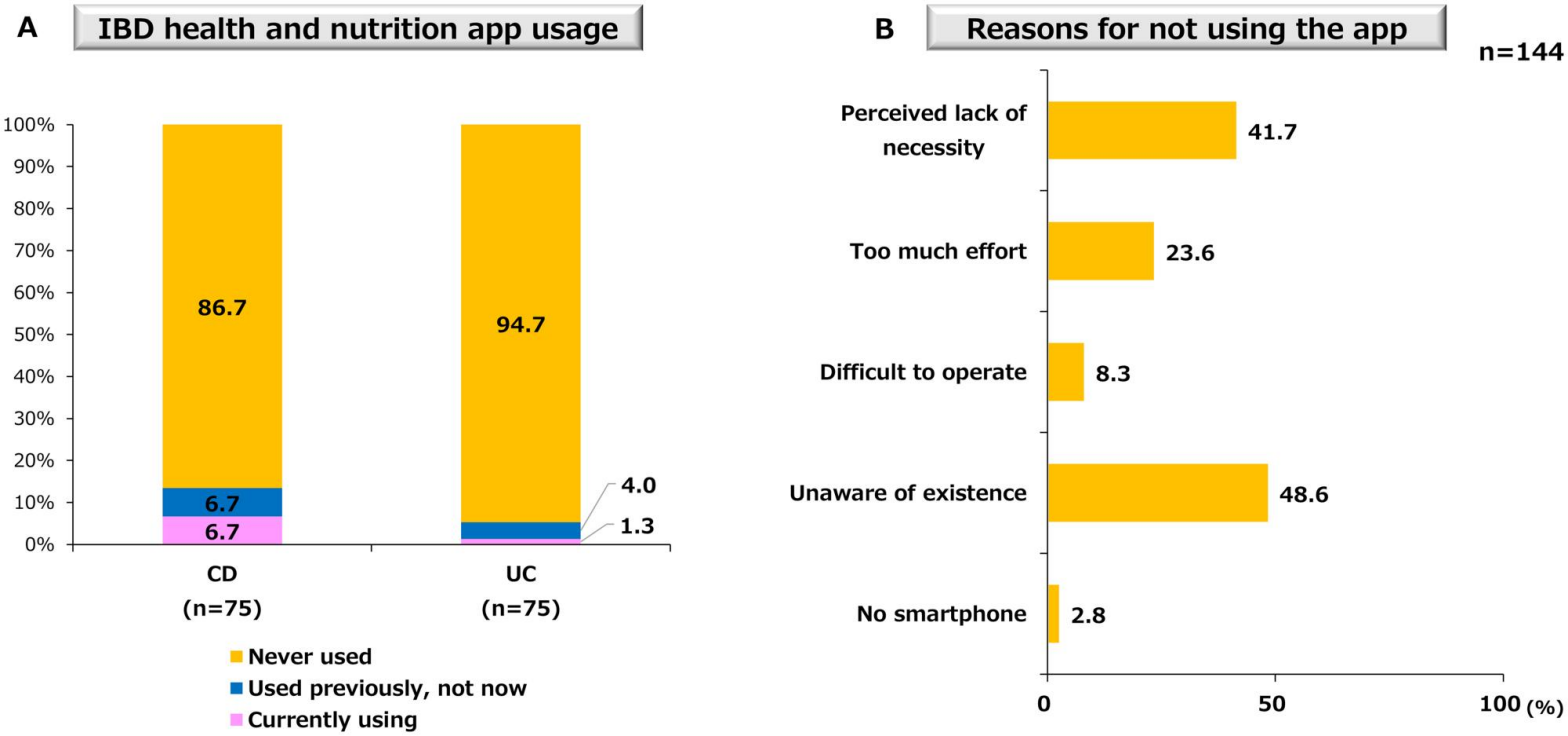
Utilization of health and nutrition management applications for IBD. (A) Experience with IBD‑related health/nutrition apps by disease group (CD vs UC), categorized as currently using, used previously but not now, and never used. (B) Reasons for not using apps among patients not currently using apps (*n* = 144), including unaware of existence, perceived lack of necessity, too much effort, difficulty operating, and no smartphone. Multiple responses were permitted; bars indicate the percentage endorsing each reason. Group comparisons were descriptive, and chi-square tests were applied, where appropriate. The percentages were calculated using the denominators indicated in the panel captions. CD, Crohn’s disease; IBD, inflammatory bowel disease; UC, ulcerative colitis.

Experience using health and nutrition management applications was reported by 13.3% of patients with CD and 5.3% of patients with UC, whereas daily use was reported by 6.7% and 1.3%, respectively (Figure 6A). Common reasons for nonuse included “lack of awareness” (48.6%), “perceived lack of necessity” (41.7%), and “inconvenience” (23.6%) (Figure 6B).

## Discussion

This web-based survey evaluated the implementation and continuation of nutritional therapy, self-perceived relapse triggers, awareness of dietary management, supplement use, and utilization of health management applications among Japanese patients with IBD. IBD is a chronic condition characterized by diarrhea and abdominal pain, which significantly impairs the quality of life (QOL).^12^ Achieving remission and preventing relapse are the key goals in disease management. Although relapse rates vary depending on the definition, previous studies have consistently reported higher relapse rates in CD than in UC.^13–15^ The present study findings were consistent with these reports: 52.0% of patients with CD and 32.0% of patients with UC reported ≥1 episode of self-perceived relapse over the previous year, and patient evaluations also indicated a higher self-perceived relapse burden in CD. However, our results should be interpreted as reflecting patients’ perceived disease course, since relapse was assessed based on patient-reported symptom worsening rather than objective disease activity.

Regarding factors perceived to influence self-perceived relapse, “dietary content” was reported significantly more often by patients with CD than by patients with UC. Previous studies have shown that diet can affect relapse in IBD, and appropriate dietary management is beneficial for relapse prevention.^16^^,^^17^ Given the risk of malabsorption and increased intestinal burden, patients with CD may be more susceptible to dietary influences. Sleep disturbance has also been reported to be associated with relapse,^18^^,^^19^ and the survey revealed particularly high rates among patients with CD. Although the differences between CD and UC remain inconclusive, previous studies suggest that the impact of sleep disturbances on relapse may be greater in patients with CD.^20^ Stress has also been implicated in relapse,^21–23^ and our findings reaffirm the importance of lifestyle factors in IBD management, as both patients with CD and UC frequently report stress as an influential factor.

Nutritional therapy, along with pharmacological treatment, is considered effective for relapse prevention.^2^^,^^6^^,^^7^^,^^24^ The efficacy of ED has been well documented.^8–10^^,^^25–27^ A web-based survey conducted in 2014 using the same panel reported ED implementation rates of 44.2% for CD and 5.0% for UC,^12^ suggesting that rates have remained similar or have slightly decreased over the past decade. The growing availability of advanced therapies.^11^^,^^28^^,^^29^ may have contributed to reduced awareness of nutritional therapy. Notably, among patients with CD, the proportion of those currently taking ED was higher in the ADT group than in the non-ADT group (42.5% vs 17.1%, respectively). Although this finding should be interpreted with caution owing to the limited sample size and the likelihood that ADT users represent more refractory cases, it suggests that ED continues to be used as a complementary option even in the era in which ADT constitutes mainstream therapy. These real-world findings may reflect the clinicians’ continued perception that ED provides additive benefits to selected patients, despite advances in pharmacotherapy. Although ED is effective in combination with advanced therapies, most evidence pertains to tumor necrosis factor (TNF)-α inhibitors,^30–34^ and further studies are needed to evaluate its role alongside other advanced treatments. Beyond nutritional support, ED may exert anti-inflammatory effects through amino acids such as histidine and tryptophan^35–37^ and improve gut microbiota composition.^38^^,^^39^ In this survey, a proportion of patients reported improvements in symptoms, albumin levels, and body weight with ED use; however, continuation rates were not high, and common reasons for discontinuation included “physician’s decision” and “difficulty in intake.” These findings highlight the need to formulate improvements and strategies to support adherence. However, it should be noted that these perceived benefits were based solely on patient self-assessment and were not evaluated using objective clinical endpoints. These results should be interpreted as subjective patient experiences rather than objective evidence of the effectiveness of ED.

The awareness of dietary management was higher among patients with CD, which is consistent with previous findings.^12^^,^^40^ Supplemental use was similar in patients with CD and UC, although the clinical benefits of supplements in IBD remain unclear^41^ and warrant further investigation.

The utility of digital tools in IBD management has been increasingly recognized.^42–44^ In Japan, several applications for symptom and nutritional management are available. The overall use of health management applications was below 10% in this survey, whereas previous studies from Europe and North America have demonstrated improvements in patient engagement, medication adherence, and healthcare utilization with the use of digital and e‑health applications as part of IBD care strategies. In this context, the low adoption observed in our cohort suggests a gap between the evidence supporting these tools and their real‑world implementation,^42–44^ and the use of health management applications needs to be encouraged in Japan.

This study has several limitations. First, as an Internet-based survey, a selection bias could not be excluded. In particular, the proportion of male participants was relatively high in this survey. Although the male-to-female ratio observed in patients with CD was broadly consistent with recent nationwide epidemiological data from Japan,^45^ the proportion of male participants among patients with UC was higher than that reported in population-based studies, suggesting a potential selection bias in the UC cohort. Such discrepancies in sex distribution may reflect differences in study design and recruitment methods. Previous hospital-based surveys, registry studies, and web-based questionnaire studies have shown that participant characteristics may vary depending on data source and participation behavior, particularly in patient-reported research.^46–49^ Second, as this was a questionnaire-based study, the responses relied on self-reporting, which may have introduced inaccuracies and recall bias regarding self-perceived relapse frequency and treatment adherence. In particular, self-perceived relapse frequency was based on a self-reported symptomatic relapse over the preceding 12 months, rather than a standardized clinical definition based on biomarkers, endoscopic findings, or physician-assessed disease activity. Therefore, the self-perceived relapse frequency in this survey may capture heterogeneous events, including transient symptom fluctuations that do not necessarily reflect objective inflammatory activity, while previous studies have demonstrated reasonable concordance between web-based patient-reported data and physician assessments.^50^^,^^51^ However, this study could not validate the responses against clinical records. Third, the sample size was relatively small (150 cases), which limits generalizability. This is due to the estimated feasible recruitment from the survey panel, with equal targets for CD and UC. Future studies should aim for larger sample sizes and use broader recruitment strategies. Despite these limitations, this survey provided valuable insights into the current status of nutritional therapy, self-perceived relapse perception, dietary management awareness, supplement use, and digital tool utilization among Japanese patients with IBD.

In conclusion, nutritional therapy in Japanese patients with IBD is primarily implemented for CD, and its clinical relevance in CD management has been suggested. Future efforts should focus on developing specific dietary guidelines, supporting continued ED use, and promoting the use of digital health tools.

## Data Availability

The data used and analyzed in this study are not publicly available but are available from the corresponding author upon reasonable request.
